# Role of AI in diagnostic imaging error reduction

**DOI:** 10.3389/fvets.2024.1437284

**Published:** 2024-08-30

**Authors:** Silvia Burti, Alessandro Zotti, Tommaso Banzato

**Affiliations:** Department of Animal Medicine, Production and Health, University of Padua, Padua, Italy

**Keywords:** artificial intelligence, error, machine learning, image quality, radiology—education

## Abstract

The topic of diagnostic imaging error and the tools and strategies for error mitigation are poorly investigated in veterinary medicine. The increasing popularity of diagnostic imaging and the high demand for teleradiology make mitigating diagnostic imaging errors paramount in high-quality services. The different sources of error have been thoroughly investigated in human medicine, and the use of AI-based products is advocated as one of the most promising strategies for error mitigation. At present, AI is still an emerging technology in veterinary medicine and, as such, is raising increasing interest among in board-certified radiologists and general practitioners alike. In this perspective article, the role of AI in mitigating different types of errors, as classified in the human literature, is presented and discussed. Furthermore, some of the weaknesses specific to the veterinary world, such as the absence of a regulatory agency for admitting medical devices to the market, are also discussed.

## 1 Introduction

The topic of error mitigation in diagnostic imaging is a relatively unexplored field in the veterinary literature. Indeed, to the best of the authors’ knowledge, only two papers investigating such a topic are available (1, 2). Likewise, incidence rates and the overall costs associated with diagnostic imaging errors have been poorly investigated in veterinary medical practice. Indeed, only one study (3) reports the radiologic error rate being comparable to what is reported in human medicine. Instead, an entire set of literature devoted to analyzing the most common causes of diagnostic imaging errors, along with possible solutions, is currently available in human medicine (4, 5). It is important to understand that diagnostic imaging errors are much more intricate than they might seem because they involve a complex interaction between individual psychological (6) environmental, and educational factors (7). A diagnostic error is defined as a “deviation from the expected norm” (8), and the consequences for the patient may vary from no consequences to death. Renfrew et al. (9) first proposed a comprehensive classification of the causes of diagnostic imaging errors, which were subsequently modified by Kim and Mansfield (10). In addition, some authors have approached this complex theme from different perspectives, ranging from the identification of different cognitive biases (6), to the analysis of interpretative errors (4), to the strategies for error reduction (11).

It is important to note at this point that a universally recognized “etiology” of errors in human diagnostic imaging is currently unavailable, and the definitions and the solutions proposed for different scenarios may vary among authors. In recent years, we have witnessed an increased interest in the applications of AI in the veterinary diagnostic imaging field (12, 13). Among other applications, AI is mainly used as a supportive tool to guide the interpretation of medical images in veterinary medicine. Even if AI is reported to have an overall lower error rate than radiologists have both in human (14) and veterinary medicine (15), dealing with such a technology is not as straightforward as it might seem (16, 17). This perspective analysis aims to examine the role of AI in mitigating each source of error in veterinary imaging through the error classification suggested by Kim and Mansfield (10).

## 2 Types of errors and role of AI in mitigation

### 2.1 Complacency

“Complacency refers to over-reading and misinterpretation of findings, a finding is detected but attributed to the wrong cause (false positive-error)” (10). This type of error is reported to be uncommon (0.9%) in human medicine, whilst no data are available in veterinary medicine. In this latter field, a discrepancy between the AI system output and the radiologist’s interpretation is likely to occur. AI systems are reported to generate lower error rates (including both false positives and false negatives) than radiologists (at least for some specific findings) (15). Veterinary radiologists should therefore consider reinterpreting findings, taking the AI results into account.

### 2.2 Faulty reasoning

“Error of over-reading and misinterpretation, in which a finding is appreciated and interpreted as abnormal but is attributed to the wrong cause. Misleading information and a limited differential diagnosis are included in this category” (10). At present, the available AI systems only detect specific radiographic findings (15, 18, 19) and are not able to provide differential diagnoses based on the clinical findings. Large language models (LLMs) (20) capable of interpreting the images and generating a list of differentials based on the medical history will soon be available, thus potentially reducing this type of error.

### 2.3 Lack of knowledge

“The finding is seen but is attributed to the wrong cause because of a lack of knowledge on the part of the viewer or interpreter” (10). This type of error is, to the authors’ knowledge, particularly relevant in the veterinary scenario, where most radiographic images are not interpreted by a radiologist but by general practitioners. As mentioned earlier, current AI-based systems cannot correlate the imaging findings with a specific list of differentials based on the medical history and therefore, to date, AI has had limited impact in mitigating this type of error.

### 2.4 Under-reading

“The lesion is not detected.” According to Kim and Mansfield (10), this alone accounts for 42% of the total diagnostic errors. Under-reading is, most likely, one of the main reasons for implementing AI systems in the day-to-day routine. Indeed, under-reading stands as a very common problem that might arise from both individual and environmental situations (7). The role of AI in mitigating this type of error is, potentially, a game changer as AI systems are not subjected to cognitive biases or environmental contexts (overworking, challenging working environment, distractions, etc.). On the other hand, the final user needs to consider that AI system accuracy is also affected by several factors, such as image quality or lesion rate in the database (21). Lastly, the user needs to be aware that most of the veterinary AI-based systems have a variable reported accuracy in the detection of specific lesions. For instance, accuracy in detecting pleural effusion is usually very high (15, 18, 22) whereas accuracy for pulmonary nodules or masses is significantly lower (18, 23).

### 2.5 Poor communication

“The lesion is identified and interpreted correctly, but the message fails to reach the clinician.” Reliable communication of imaging findings is vital for the correct management of patients, both in veterinary and human medicine. Imaging reports use highly specialized terminology, and the accurate interpretation of these terms relies on the expertise of the referring clinician. This type of error is reported to be quite rare (10) as, when a report is unclear, a direct explanation is usually required from the reporting physician. To this end, incorporating large language models (LLMs) (20) within the reporting systems could help in creating more homogeneous reports and therefore improve communication between the clinician and the radiologist.

### 2.6 Prior examination/history

“The finding is missed because of failure to consult prior radiologic studies or reports” and “The finding is missed because of the acquisition of inaccurate or incomplete clinical history.” These are among the most common types of errors, and the American College of Radiology recommends that all the patients’ previous reports should be available to the radiologist during exam evaluation (10). This type of error is most relevant in teleradiology services since most of these services do not have access to complete patient history. AI-based products guiding radiologists (both in human and veterinary medicine) throughout the reporting process (from image acquisition to final report) could be important in mitigating these errors. For example, using LLMs to promptly summarize the patient’s clinical history could provide the radiologist with quick and useful information.

### 2.7 Location

“The finding is missed because the location of a lesion is outside the area of interest on an image, such as in the corner of an image.” These errors are fairly common and are possibly related to what is referred to as “intentional” or “tunnel vision bias” (10). These are well-known cognitive biases. In a famous experiment, radiologists were asked to detect pulmonary nodules from CT images. The picture of a gorilla, 10 times larger than the average nodule, was placed in one of the CT images. Surprisingly, 83% of the radiologists did not report seeing the gorilla, despite eye-tracking technologies demonstrating that all the radiologists looked at it (24). In this case, using AI systems to assist the radiologist could help in reducing these types of errors provided that the AI systems themselves do not generate numerous false positives (16). Indeed, as demonstrated by Bernstein et al. (16), a faulty AI decreases radiographers’ accuracy especially if the results of the AI are shown in the final report.

### 2.8 Satisfaction of search

“The finding is missed because of the failure to continue to search for additional abnormalities after a first abnormality is found.” This is a common situation, especially when advanced imaging modalities, such as CT or MRI, are evaluated (10). To the best of the authors’ knowledge, no algorithm for lesion detection in advanced imaging modalities (CT or MRI) has been proposed in the veterinary literature, and, therefore, the usefulness of AI in the reduction of such an error has yet to be established.

### 2.9 Complication

“Complication from a procedure,” meaning untoward events that could happen during an invasive examination procedure (9). This is reported to be an uncommon type of error in human medicine (10). The role of AI in the reduction of such an error is similar to that regarding other error types (e.g., prior examination).

### 2.10 Satisfaction of report

“The finding was missed because of the complacency of the report, and over-reliance on the radiology report of the previous examinations.” This type of error arises from what is known as alliterative bias, meaning that one radiologist’s judgement is influenced by that of another radiologist. To avoid this sort of bias (6), suggest that the radiologist should read previous reports only after rendering the interpretation of findings. This is one of the most dangerous types of errors, as it is perpetuated from one study to the next (10). The authors believe that AI could play a prominent role in reducing these error types. In fact, AI systems are unaware of the results of prior studies and could therefore help the clinician make more factual-based decisions that are devoid of cognitive biases.

## 3 Conclusion

A summary of the error types according to Kim and Mansfield (10) and the possible contribution of AI-based products in their mitigation is reported in Table 1. AI is still a very young technology in veterinary medicine and, despite the increasing number of applications available on the market, is far from being part of most practices’ clinical routine. The same is also true, to some extent, in human medicine. Indeed, despite the large investments in and the media impact of AI, the diffusion of AI-based systems is still limited, and actual improvements in healthcare quality related to the widespread adoption of these technologies are still to be demonstrated (25).

**Table 1:** Possible errors according to Kim and Mansfield (10) and role of AI in mitigation.

Type of error	Role of AI
Complacency	Yields lower number of false positives than radiologists do
Faulty reasoning	Limited usefulness of AI. Education plays a larger role in mitigating this error type
Lack of knowledge	Limited usefulness of AI (LLMs might be more effective)
Under-reading	Varies depending on the accuracy for each specific finding
Poor communication	Limited usefulness. LLMs could provide a means to homogenize the reports
Prior examination/history	AI-assisted reporting and AI-based tools to create quick summaries of the clinical history could help in mitigating this type of error
Location	AI scans the entire image/scan and is not influenced by the position of the lesion
Satisfaction of search	AI is unaware of the reasons for the scan/image and checks the entire exam
Complication	Similar to prior examination
Satisfaction of report	AI-based products are not influenced by this error type and could help in taking more factual-based decisions

LLMs, large language models.

It is the authors’ opinion that AI will likely have different impacts on human and veterinary diagnostic imaging, mostly due to the intrinsic differences that exist between these two disciplines. The number of board-certified radiologists in veterinary medicine is still limited compared to those in human medicine, and therefore it is common practice for veterinary diagnostic images to be interpreted by non-specialists. This poses some questions regarding the effectiveness of these AI-based computer-aided systems in veterinary medicine. In fact, it is reported that AI has a variable accuracy for different radiographic findings (18, 23). If the operator cannot determine the accuracy of the AI system’s findings, relying on these systems might lead to misleading outcomes.

In the perspective article presented here, we did not address the importance of AI algorithms in assessing the quality of medical images. This application has been scarcely explored in veterinary medicine, and to date, only two studies highlights these algorithms as a promising tool to enhance the accuracy of interpreting canine radiographs by identifying technical errors (26, 27). Conversely, in human medicine, numerous AI-based algorithms have been developed for evaluating the quality of chest X-ray images, showing promising results (28, 29). This is a field where AI algorithms could again contribute to reducing radiologists’ interpretative error rates by automatically screening the quality of diagnostic images before interpretation, similar to what is already happening in human medicine.

In human medicine, new medical devices need to be approved by a regulatory agency, such as the European Medicines Agency in Europe or the Food and Drug Administration in the United States (30). In veterinary medicine, such a regulatory agency does not exist and therefore, to date, there has not been a way to certify vendors’ claims regarding the accuracy and stability of the proposed systems (31). It is the authors’ opinion that, in such a scenario in veterinary medicine, correct and impartial information to the final users is of vital importance in order to avoid misuse and possible fraud.

## Data availability statement

The original contributions presented in the study are included in the article/supplementary material, further inquiries can be directed to the corresponding author.

## Author contributions

SB: Writing – review & editing. AZ: Writing – review & editing. TB: Writing – original draft, Writing – review & editing.
